# Associations of antenatal care visit with utilization of institutional delivery care services in Afghanistan: intersections of education, wealth, and household decision-making autonomy

**DOI:** 10.1186/s12884-022-04588-0

**Published:** 2022-03-26

**Authors:** Mostafizur Rahman, Priom Saha, Jalal Uddin

**Affiliations:** 1 Department of Science and Humanities, Bangabandhu Sheikh Mujibur Rahman Aviation and Aerospace University, Old Airport, Tejgaon, Dhaka, 1215 Bangladesh; 2 Institute of Statistical Research and Training, University of Dhaka, Dhaka, 1000 Bangladesh; 3 Department of Community Health and Epidemiology, Dalhousie University, Halifax, Nova Scotia Canada

**Keywords:** Maternal health, Antenatal care (ANC) visit, Institutional delivery care service, Delivery at a health facility, Delivery assisted by a skilled birth attendant

## Abstract

**Background:**

The importance of antenatal care (ANC) visits in safe motherhood and childbirth is well-documented. However, less is known how social determinants of health interact with ANC visits in shaping the uptake of professional delivery care services in low-income countries. This study examines the associations of ANC visits with institutional delivery care utilization outcomes in Afghanistan. Further, we assess the extent to which ANC visits intersect with education, wealth, and household decision-making autonomy in predicting two outcomes of delivery care utilization- delivery at a health facility and delivery assisted by a skilled birth attendant.

**Methods:**

We used data from the Afghanistan Demographic and Health Survey (AfDHS) 2015. The analytic sample included 15,590 women of reproductive age (15–49). We assessed the associations using logistic regression models, estimated the predicted probability of delivery care outcomes using statistical interactions, and presented estimates in margins plot.

**Results:**

Multivariable adjusted analyses suggest that women who had 4 or more ANC visits were 5.7 times (95% CI = 4.78, 7.11; *P* < 0.05) more likely to use delivery care at a health facility and 6.5 times (95% CI = 5.23, 8.03; *P* < 0.05) more likely to have a delivery assisted by a skilled birth attendant compared to women who had no ANC visit. Estimates from models with statistical interactions of ANC visits with education, wealth, and decision-making autonomy suggest that women with higher social status were more advantageous in utilizing institutional delivery care services compared to women with lower levels of social status.

**Conclusion:**

Our findings suggest that the associations of ANC visits with institutional delivery care services are stronger among women with higher social status. The results have implications for promoting safe motherhood and childbirth through improving women’s social status.

**Supplementary Information:**

The online version contains supplementary material available at 10.1186/s12884-022-04588-0.

## Introduction

Sustainable Development Goal (SDG) 3.1 aims at reducing maternal mortality to less than 70 deaths per 100,000 live births by 2030 [1]. Worldwide, 295,000 women (or 211 per 100,000) died due to pregnancy-related complications in 2017. More specifically, 810 women died every day in 2017 and 94% of these deaths occurred in low and lower-middle-income settings. In Afghanistan, the maternal mortality rate prevails at 638 per 100,000 live births [2], which is far from achieving the SDG target. In reducing maternal mortality, the World Health Organization [3] advocated that delivery at a health facility and delivery assisted by a skilled birth attendant are the foremost priorities.

Antenatal care (ANC) visits and delivery care services play a crutial role in ensuring safe motherhood. The use of ANC services helps pregnant women to interact with the healthcare system. ANC visits typically help diagnose and treat pregnancy-related complications, facilitate use of delivery care services, and provide pregnant women with a broad range of health promotion and preventive services. The World Health Organization and policy guidelines in Afghanistan recommend receiving at least 4 ANC visits during pregnancy [4]. However, the uptake of ANC services remains substantially low in Afghanistan, where only 21% of women had four or more ANC visits in 2018 [4]. ANC visits during pregnancy can significantly promote the continuation of institutional care for safe childbirth and postnatal care. Higher ANC visits can reduce the number of deaths by preventing pregnancy-related complications [2, 5]. Conversely, lower ANC visits and utilization of ANC services increase the risk of maternal mortality by double [6].

Socioeconomic status (SES) is a significant predictor of the utilization of institutional maternity care services. Higher socioeconomic status helps increase awareness of seeking safer maternity care and provides the financial and material resource base necessary for uptaking institutional delivery care services [7–9]. Multiple SES indicators, including the mother’s education, wealth, and place of residence, can significantly shape delivery care services. For instance, the 2018 Afghanistan Health Survey suggests that most women who delivered child at home were from rural areas and with lower levels of wealth and education. The home births are usually attended by a friend, neighbor, relative, or traditional birth attendant. On the contrary, women receiving delivery care assisted by a skilled birth attendant mainly were from urban and upper social strata [4]. Likewise, several studies have found that SES, including wealth, education, residence, and decision-making autonomy, were significant predictors of the uptake of the institutional delivery care services and having birth assisted by a skilled birth attendant in Afghanistan [10–14].

Existing studies focusing on socioeconomic disparities in institutional delivery care services employ a unidimensional and additive approach implying that social status and structural factors are mutually separate and are additive determinants of social disparity in health services utilization [15–18]. These studies fail to grasp the intersecting nature of social status and structural elements with an additive analytic approach. Intersectionality literature has powerfully shown that social status indicators may intersect and produce unique social (dis)advantage and opportunity structure [19–21]. The intersectionality perspective demonstrates how social identity, social position, cultural norms, geography, and opportunities may produce interlocking and mutually reinforcing effects on health services utilization [22]. In essence, the intersectionality approach advocates for examining how multiple social categories may explain health disparities in a joint, synergistic, and multiplicative way [9, 23]. These synergistic and multiplicative effects in quantitative studies are often evaluated using statistical interactions or multilevel analyses.

In line with the intersectionality perspective, we hypothesize that women with lower education, lower wealth, and diminished autonomy in household decision-making constitute a population group with ‘multiple jeopardies’ or ‘disadvantages’ [24, 25]. More specifically, multiple social disadvantages, including poorer wealth base, education, autonomy, and regional contexts, may intersect with one another to produce inequality in necessary health services utilization. Although the intersectionality approach has been widely used in population health studies, mostly in the context of industrially developed countries, this approach has rarely been used in health services research in low-income settings. An investigation of examining the inequality in health services research using an intersectionality perspective has implications for informing interventions and public health programs aiming to improve maternal and child healthcare services use in low-income countries. Applying an intersectionality perspective in health services research and leveraging the Demographic and Health Survey of Afghanistan, we examined the intersecting effects of ANC visits, women’s education, household wealth, and household decision-making autonomy on two outcomes of institutional delivery care services utilization in Afghanistan - delivery at a health facility and delivery assisted by a skilled birth attendant.

## Data and methods

### Data

**Fig. 1:**
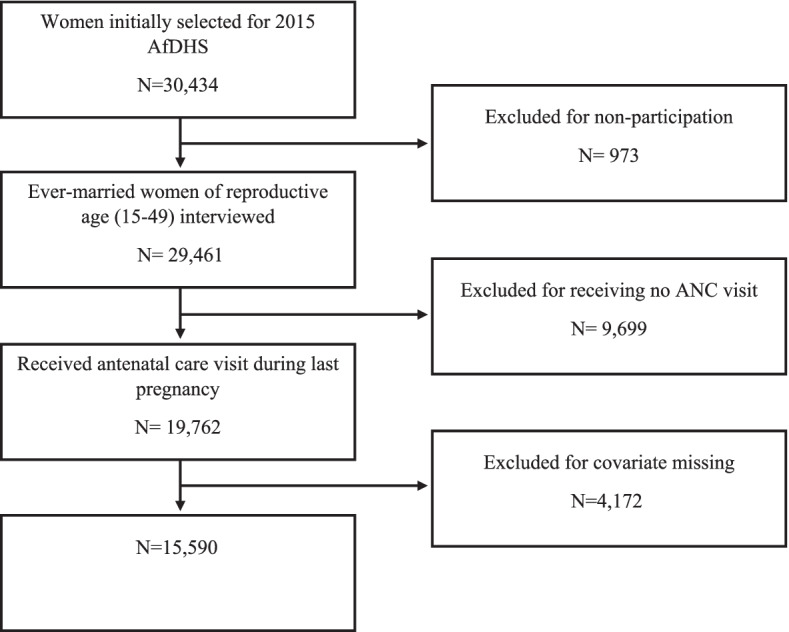
Flowchart of sample selection criteria

We analyzed de-identified data from the 2015 Afghanistan Demographic and Health Survey (AfDHS). The AfDHS is a nationally representative cross-sectional household survey, which used a two-stage stratified sampling technique to collect data on nutrition, mortality, fertility, maternal, and reproductive health of ever-married women. For the survey, 25,741 households were initially selected, and 24,395 of them were finally interviewed. The response rate was 98%. Data were collected between June 15, 2015 and February 23, 2016. Data entry was done twice for 100% accuracy and verification. In this study, the analysis was limited to the women reporting most recent singleton live birth. After excluding observations with missing values, the final analytic sample included 15,590 women aged between 15 and 49 years and is shown in Fig. 1.

### Outcome variables

The analysis included two binary outcomes in this paper – delivery at a health facility and delivery assisted by a skilled birth attendant. Delivery at a health facility was coded 1 if the delivery took place at a health facility (e.g., government or private hospital/health center/health post, maternal and child welfare center, NGO static clinic, and sub-district health center) and 0 if delivery was at home. Likewise, delivery assisted by a skilled birth attendant was coded 1 if a skilled birth attendee assisted the delivery (e.g., doctor, nurse, community skilled birth attendant, family welfare visitor, and midwife) and 0 otherwise.

### Key explanatory variables

ANC visit: The key exposure variable in the analysis was ANC visits during pregnancy. Women were asked about the number of their ANC visits during pregnancy. The response was continuous and ranged between 0 and 20. The following categories were used in the analysis: 0, 1, 2, 3, and ≥ 4 ANC visits.

Covariates: The analysis adjusted for women’s demographic factors, socioeconomic factors, and women’s autonomy index. Demographic variables included women’s current age (in years) and place of residence (rural/urban). Socioeconomic variables included education (in years), wealth index, and current employment (employed/not employed), while other covariates were decision-making autonomy and beating wife not justified index.

Wealth index: Wealth index is a measure of a cumulative living standard of a household. This index consists of the household’s possession of various household assets, dwelling characteristics, and farm characteristics. For instance, assets and dwelling characteristics include radio, television, bicycles, mobile phones, computers, refrigerators, housing construction materials, furniture, farm animals, agricultural land, sanitation facilities, water access, etc. Using the principal component analysis, households were assigned scores based on their ownership of type and amount of the above assets and household items. Finally, a continuous asset score was assigned to each household, and then they were categorized into five wealth quintiles [26].

Decision-making autonomy index: Decision-making autonomy index was a composite measure of three household decision-making questions, including a) “Person who usually decides on respondent’s health care”, b) “Person who usually decides on large household purchases”, and c) “Person who usually decides on visits to family or relatives”. For this index, the response “respondent alone” was coded 2, “respondent and husband/partner” was coded 1, and other responses such as “respondent and other person”, “husband/partner alone”, and “someone else” were coded 0. The summed score of three items ranged from 0 to 6, where a higher score referred to women’s higher autonomy in the household decision-making process.

 Beating wife not justified index: Beating wife not justified was a summative index composed of the following five items representing women’s perception about violence perpetrated by their husbands/partners against them [27]: a) “Beating justified if wife goes out without telling husband”, b) “Beating justified if wife neglects the children”, c) “Beating justified if wife argues with husband”, d) “Beating justified if wife refuses to have sex with husband”, and e) “Beating justified if wife burns the food”. The response “no” to all these items was coded 0, and the response “yes” was coded 1, while the response “don’t know” was coded missing. Then, the scores of all items were summed, and the total score ranged between 0 and 5, where score 0 indicated beating not justified, and score 1–5 indicated that women perceived the beating as justified.

### Statistical analyses

In the beginning, we presented sample characteristics of the respondents. Then, we assessed the associations between ANC visits and two outcomes, using logistic regression analysis adjusting for the potential covariates. In regression analysis, we estimated three models. We minimally adjusted for respondents’ age and place of residence in the first model. The second model further adjusted for socioeconomic factors, including education (in years), wealth index, and employment status. In the third model, we further adjusted for women’s household decision-making autonomy and an index of beating wife not justified in addition to the covariates adjusted in the second model. We estimated model fit statistics using Akaike information criterion (AIC) and Bayesian information criterion (BIC). The model with minimum AIC and BIC values was best fit and selected for final regression. The regression analysis also incorporated complex survey design factors, including survey strata, clusters, and weights.

**Fig. 2:**
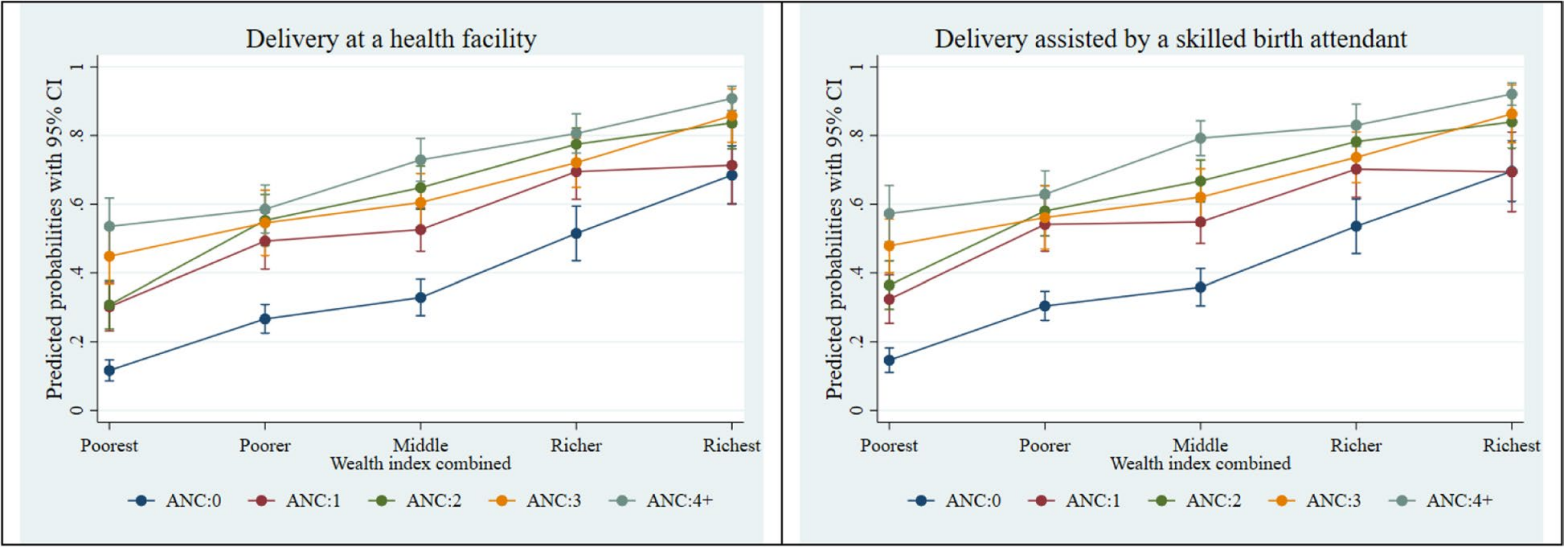
Adjusted predictions for institutional delivery care utilization by ANC visits by wealth index. CI, confidence interval

**Fig. 3:**
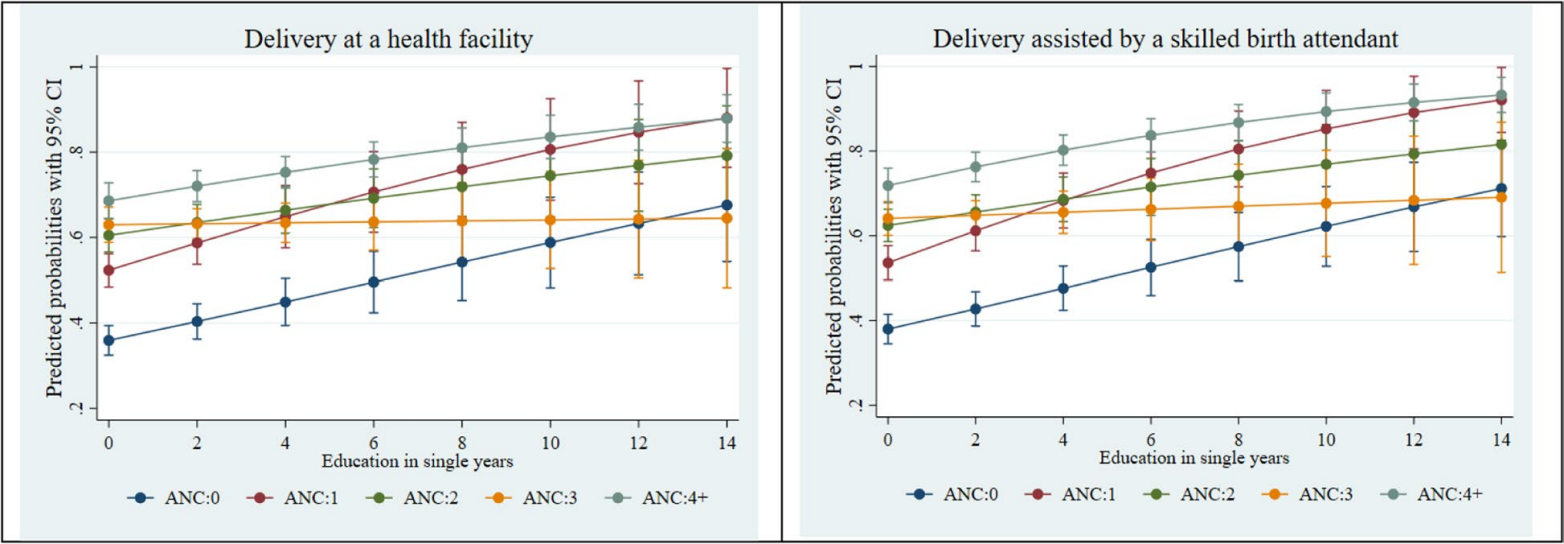
Adjusted predictions for institutional delivery care utilization by ANC visits by education. CI, confidence interval

**Fig. 4:**
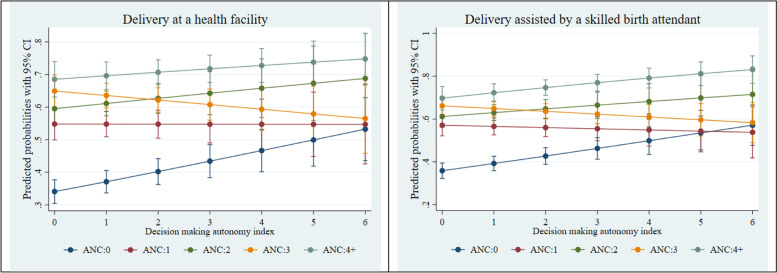
Adjusted predictions for institutional delivery care utilization by ANC visits by decision-making autonomy index. CI, confidence interval

**Fig. 5:**
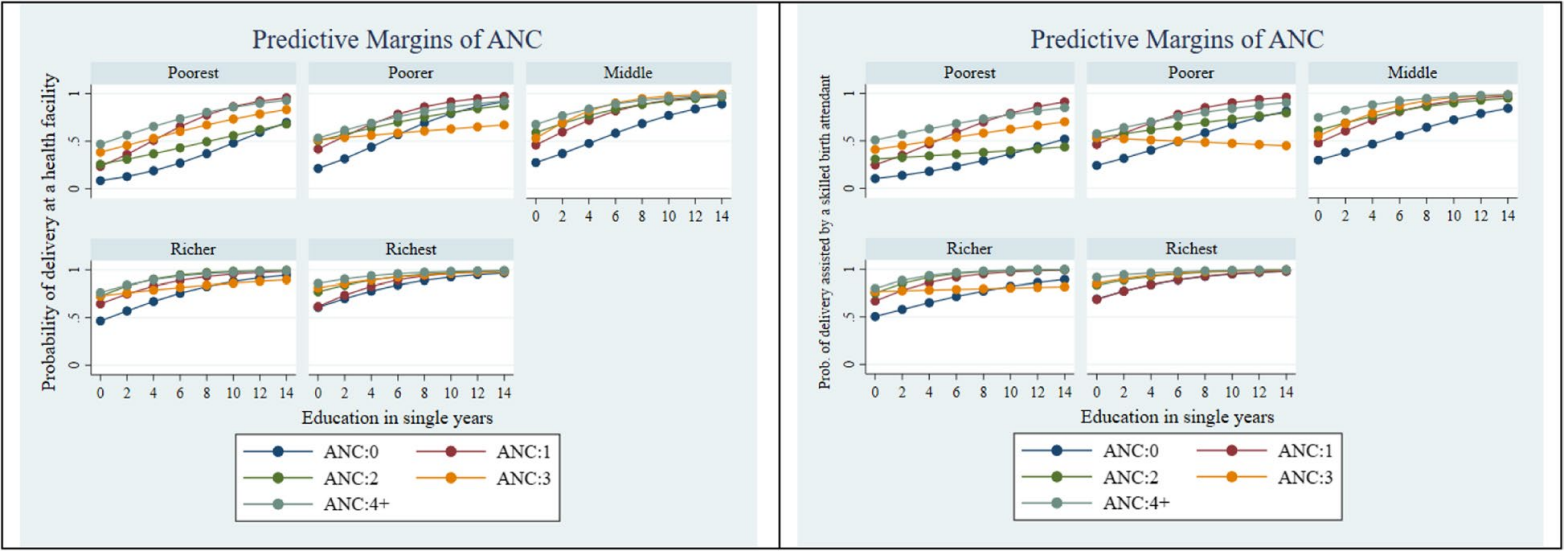
Adjusted predictions for institutional delivery care utilization by ANC visits by wealth index by education in years

The intersecting effects of ANC visits with SES factors and household decision-making autonomy were examined using a series of two-way and three-way statistical interactions. We used Stata’s margins command (at means) to compute adjusted predicted probabilities from the models with interactions for ease of interpretation. We presented the adjusted predicted probabilities in Figs. 2, 3, 4 and 5. The formal test result of interaction is presented in Supplementary Table 1. Stata 15.1 (Stata Corp LP, College Station, TX) was used for all data analyses.

Additionally, a supplementary analysis examined the relationships using a multilevel approach as the AfDHS 2015 followed a multi-stage sampling in which households (level-1) are nested in the regions (level 2). To capture variability across regions in Afghanistan, a multilevel generalized linear regression analysis with logit link and binomial family was performed.

## Results

Table 1 shows the characteristics of the study sample by antenatal care (ANC) visits in Afghanistan. Among women with 4 or more ANC visits, only 12% were from the lowest wealth quintile, and 29% were from the richest wealth quintile. Women with the highest years of education (2.53) had 4 or more ANC visits than the lowest-educated women (0.53) with no ANC visit. The average age of the respondent was 29 years across categories of ANC visits. Women had higher decision-making autonomy scores in the 4 or more ANC visits category than other ANC visits categories. For instance, the average decision-making autonomy score was 1.27 for women with no ANC visits and 1.87 for women with 4 or more ANC visits. Rural women tend to have a lower number of ANC visits. For example, of the women with no ANC visits, 82% reside in the rural area. On the contrary, 58% of the women with 4 or more ANC visits are urban residents.

**Table 1:** Sample characteristics of women by ANC visit (*N* = 15,590)

Variables, % (N)	Antenatal Care (ANC) Visits						χ^2^/F statistic (*P* value)
	0 Visit	1 Visit	2 Visits	3 Visits	≥4 Visits	Total	
Wealth Index							
Poorest	0.21 (1421)	0.19 (319)	0.19 (490)	0.19 (342)	0.12 (334)	0.19 (2906)	847.94 (< 0.01)
Poorer	0.27 (1825)	0.24 (407)	0.21 (558)	0.18 (312)	0.14 (369)	0.22 (3471)	
Middle	0.24 (1600)	0.24 (420)	0.22 (584)	0.20 (351)	0.20 (523)	0.22 (3478)	
Richer	0.19 (1303)	0.22 (370)	0.24 (627)	0.25 (442)	0.25 (671)	0.22 (3413)	
Richest	0.09 (639)	0.12 (205)	0.14 (365)	0.19 (332)	0.29 (781)	0.15 (2322)	
Education (in years), mean (SD)	0.53 (0.02)	0.85 (0.06)	1.19 (0.06)	1.70 (0.08)	2.53 (0.07)	1.14 (0.02)	970.28 (< 0.01)
Age (in years), mean (SD)	29.00 (0.08)	28.93 (0.16)	28.53 (0.13)	28.86 (0.16)	28.78 (0.13)	28.89 (0.05)	3.00 (0.0173)
Decision-making autonomy, mean (SD)	1.27 (0.01)	1.31 (0.03)	1.46 (0.03)	1.60 (0.03)	1.87 (0.03)	1.50 (0.01)	91.88 (< 0.01)
Current employment							
Not employed	0.83 (5664)	0.87 (1496)	0.90 (2367)	0.90 (1595)	0.90 (2420)	0.86 (13545)	136.31 (< 0.01)
Employed	0.17 (1124)	0.13 (225)	0.10 (257)	0.10 (184)	0.10 (258)	0.14 (2048)	
Place of residence							
Urban	0.18 (1068)	0.21 (383)	0.17 (459)	0.27 (538)	0.42 (1242)	0.24 (3690)	725.28 (< 0.001)
Rural	0.82 (4775)	0.79 (1448)	0.83 (2276)	0.73 (1462)	0.58 (1732)	0.76 (12000)	
Beating wife not justified							
Justified	0.83 (5654)	0.84 (1453)	0.86 (2249)	0.87 (1547)	0.79 (2340)	0.84 (13059)	43.19 (< 0.001)
Not justified	0.17 (1134)	0.16 (268)	0.14 (375)	0.13 (232)	0.20 (522)	0.16 (2531)	
Total	6788 (43.50)	1721 (11.04)	2624 (16.83)	1779 (11.41)	2678 (17.18)	15,590 (100)	

Chi-square for categorical and F-statistic for continuous variables

Table 2 reports the distribution of delivery at a health facility and delivery assisted by a skilled birth attendant by ANC visit categories and associated chi-square test results. Nearly 27% of women with 4 or more ANC visits had a delivery at a health facility and had a skilled birth attendant during delivery. The associations of ANC visits with delivery at a health facility and delivery assisted by a skilled birth attendant were significant at 0.001% level of significance.

**Table 2:** Chi-square test of ANC visit, delivery at a health facility, and delivery assisted by a skilled birth attendant

Number of ANC visits	Delivery at a health facility		Delivery assisted by a skilled birth attendant	
	% (N)	χ^2^	% (N)	χ^2^
0 visit	23.87 (1851)	2700**	23.97 (1911)	2900**
1 visit	11.72 (909)		11.69 (932)	
2 visits	21.50 (1667)		24.45 (1710)	
3 visits	15.97 (1238)		15.77 (1257)	
≥4 visits	26.94 (2089)		27.11 (2161)	

**p < 0.001

Table 3 presents the adjusted odds ratio (OR) with 95% confidence interval (CI) from the logistic regression analysis. After adjustment for potential covariates in all models, we found that number of ANC visit was significantly associated with increased odds of delivery at a health facility and delivery assisted by a skilled birth attendant. In fully-adjusted model 3, the odds of delivery at a health facility were greater for those who had 4 or more ANC visits (OR = 5.74; 95% CI = 4.78, 7.11; *P* < 0.05) than those who had no ANC visit. Likewise, the odds of delivery assisted by a skilled birth attendant were higher at higher levels of ANC visit. The odds of having a delivery assisted by a skilled birth attendant were 6.48 (95% CI = 5.23, 8.03; *P* < 0.05) for women who had 4 or more ANC visits compared to those who had no ANC visits. Overall, the results suggest that women with a higher number of ANC visits had greater odds of having institutional delivery care services.

**Table 3:** Adjusted odds ratios and 95% confidence intervals for institutional delivery care indicators (*N* = 15,590)

Number of ANC visits (Ref: 0)	Delivery at a health facility			Delivery assisted by a skilled birth attendant		
	OR (95% CI)	OR (95% CI)	OR (95% CI)	OR (95% CI)	OR (95% CI)	OR (95% CI)
	Model 1	Model 2	Model 3	Model 1	Model 2	Model 3
1	2.41* (1.96,2.97)	2.47* (1.92,3.01)	2.45* (1.96,3.03)	2.38* (1.92,2.94)	2.39* (1.92,2.97)	2.38* (1.91,2.97)
2	3.64* (2.92,4.53)	3.61* (2.93,4.43)	3.57* (2.92,4.37)	3.66* (2.94,4.55)	3.61* (2.93,4.43)	3.58* (2.92,4.39)
3	4.11* (3.25,5.21)	3.88* (3.20,4.77)	3.84* (3.06,4.69)	4.07* (3.21,5.16)	3.74* (3.02,3.65)	3.71* (2.98,4.62)
≥4	7.00* (5.61,8.75)	5.89* (4.75,7.38)	5.74* (4.78,7.11)	7.98* (6.28,10.06)	6.67* (5.33,8.32)	6.48* (5.23,8.03)
AIC	18,091.88	16,397.41	16,364.50	17,790.89	16,194.53	16,136.71
BIC	18,145.47	16,596.48	16,494.60	17,844.89	16,309.34	16,266.83

*P < 0.05

*ANC* Antenatal care, *CI* Confidence interval, *OR* Odds ratio

Model 1: Adjusted for respondents’ current age and place of residence

Model 2: Further adjusted for education, wealth, and current employment status

Model 3: Further adjusted for decision-making autonomy and beating wife not justified index

The supplementary analysis of multilevel logistic regression also finds similar directions of associations. For instance, the odds of delivery at a health facility (OR = 7.80; 95% CI = 6.53, 9.33; *P* < 0.05) and delivery assisted by a skilled birth attendant (OR = 8.90; 95% CI = 7.42, 10.69; *P* < 0.05) were greater for those who had 4 or more ANC visits than those who had no ANC visit in considering the cluster-wise variations. The cluster-level variation in the associations of categories of ANC visits with two outcomes was significantly different. The results from the multilevel analysis are presented in Supplementary Table 2.

Figures 2, 3 and 4 present the adjusted predicted probabilities obtained from a series of two-way interactions between ANC visit, wealth, education, and household decision-making autonomy, with other socio-demographic variables held at their mean. Figure 2 suggests that women who were from the richest wealth quintile had the greatest probability, among all other wealth categories, of having a delivery at a health facility and delivery assisted by a skilled birth attendant at 4 or more ANC visits. Similarly, Fig. 3 shows that as the education year increases, the probability of using institutional delivery care services (delivery at a health facility and delivery assisted by a skilled birth attendant) increases significantly for the women who have 4 or more ANC visits. Finally, Fig. 4 suggests that among women with 4 or more ANC visits, the higher the decision-making autonomy, the higher was the probability of institutional delivery care utilization- delivery at a health facility and delivery assisted by a skilled birth attendant.

Figure 5 presents the differential effect of ANC visits by years of education and wealth index with other socio-demographic variables held at their mean. The effect of ANC visits on using institutional delivery care services was generally higher among women in wealthier categories and those with higher years of education. Further, wealth quintile differences in the effect of ANC visits tend to diverge at the lower levels of education and converge or attenuate at the highest levels.

## Discussion

The number of ANC visits has been highlighted as an important parameter of improving the utilization of maternal health care. Given the importance of SES factors, many studies assessed the association of ANC visits with delivery at a health facility and delivery assisted by a skilled birth attendant using unidimensional or additive approaches, where the intersectional effects of ANC visits and socioeconomic status were absent. In this study, we found robust evidence that ANC visit intersects with SES indicators, including wealth and education, and impacts the delivery care utilization in Afghanistan.

Our analysis finds that ANC visit is an essential and significant predictor of delivery at a health facility. The results suggest that women who had four or more ANC visits were more likely to have a delivery at a health facility. Similarly, previous studies also found a positive relationship between ANC visits and delivery at a health facility [17, 18]. For instance, Berhan & Berhan [5] and Dahiru & Oche [16] reported that women who received at least four ANC visits were respectively 7 and 2 times more likely to have a delivery at a health facility. At the same range, our study found that women who received at least four ANC visits were over 5 times more likely to have childbirth at a health facility.

The analysis demonstrated that ANC visit is also a strong predictor of delivery assisted by a skilled birth attendant. More specifically, four or more ANC visits were associated with increased utilization of a delivery assisted by a skilled birth attendee. A plausible mechanism underlying this association is that women with a higher number of ANC visits have a higher awareness of pregnancy-related complications. Thus, they are more likely to seek professional delivery care services [28, 29]. So, the women who have more ANC visits at a health facility have more chances to meet skilled birth personnel and thus to have delivery there by them. Similar to our findings, prior studies have shown that having a birth assisted by a professional birth attendee is more pronounced among women who had four or more ANC visits [30–33].

It is well documented that SES indicators such as education, income, and wealth significantly shape the utilization of maternity care outcomes such as having a birth at a healthcare center [8, 16, 34] and having a delivery assisted by a skilled birth attendant [31, 32, 35–37]. This analysis found significant impacts of SES factors on the uptake of delivery care services among reproductive-aged women, similar to the prior studies. More specifically, our analysis suggests that women from socially advantageous groups (e.g., those with higher education, wealth, and autonomy) were more likely to utilize institutional delivery care services. It is well-known that women from higher SES are better equipped with financial and health-enhancing resources to use necessary services for maternity care. Broadly, women from higher social status groups are well aware, knowledgeable, health-conscious, and financially able to meet health expenditures than those from lower SES [7, 38].

In line with the intersectionality perspective, our analysis has demonstrated that the differential effect of ANC visits jointly varies by two significant SES factors – wealth index and years of education. Broadly, predicted probabilities derived from the interaction models suggest that women with higher education, wealth, and autonomy are more likely to deliver at a health facility and be assisted by a skilled birth attendant. As explained by intersectionality scholars, women with higher education, greater family wealth, and increased power in the household have multiplicative advantages and opportunities at their disposal [24, 25]. Women’s higher social standing allows them to maintain the continuity of care by establishing a usual source of care [39, 40]. Likewise, a number of intersectionality studies found similar associations between ANC visit and delivery care utilization in low-income settings [41–43]. Other studies also significantly documented that SES factors including wealth [15, 17, 44, 45], autonomy [46–48], and education [34, 48, 49] have positive effects on utilization of delivery care services. Further, our analysis indicates that predicted estimates of the associations of ANC visits with delivery care outcomes tend to be heterogeneous at the lower levels of education and converge or attenuate at the highest levels. Such findings suggest that the consequences of having multiple disadvantages put women at increased risk of not having professional maternity care services. In other words, most advantaged sections of the women tend to have better protective benefits from their relative social status.

### Strengths, limitations, and future directions

One of the strengths of this study is that it uses a nationally representative survey and can thus be inferred to the national level. We applied an intersectionality perspective, which addresses the limitation of studies that typically use an additive or unidimensional approach. In this regard, an intersectionality framework can be useful in documenting how ANC visits and various SES indicators intersect to shape the uptake of delivery care services in a low-income setting. Despite these strengths, we acknowledge a few notable limitations. The data on delivery care utilization was based on self-reports and responses that might be subject to long-recall bias and social desirability issues. To minimize the bias in our outcome variables, we limited the analysis to the last singleton live birth in the last year preceding the survey. Further, despite controlling for several potential confounding factors, we could not control for other relevant confounders such as husbands’ SES, women’s social network, and financial and physical access to healthcare centers. While existing research generally emphasizes the role of socioeconomic and demographic determinants of maternity care utilization, other factors, including healthcare provider’s poor reception, cost of institutional delivery, religious conservatism, poor transport facilities, fear of exposing private area, no permission of male partner, and fear of male birth attendant, can shape the use of institutional delivery care services [50–52]. Further, our analysis uses the number of ANC visits which cannot reflect the quality of the ANC services. The quality of ANC services may also shape the institutional delivery care services.

## Conclusion

Our analysis suggests that four or more ANC visits are significantly associated with increased institutional delivery care utilization. Further, ANC visits complexly intersect with SES indicators to predict institutional delivery care services utilization such that women from the upper social stratum enjoy multiple advantages in uptaking those services. Our findings have implications for informing interventions and targeted public health programs aiming to reduce social disparities in professional delivery care services in low-income countries.

## Supplementary Information

**Additional file 1.**

## Data Availability

Data are available online and one can access from https://dhsprogram.com.

## References

[1] United Nations (2017). Global indicator framework for the Sustainable Development Goals and targets of the 2030 Agenda for Sustainable Development Goals and targets (from the 2030 Agenda for Sustainable Development) Indicators. 48th session of the United Nations Statistical Commission.

[2] WHO, UNFPA, UNICEF, World Bank Group, The United Nations Populations (2019). Maternal mortality&nbsp;: level and trends 2000 to 2017.

[3] World Health Organization. Standards for improving quality of maternal and newborn care in health facilities. Geneva; 2016. https://cdn.who.int/media/docs/default-source/mca-documents/advisory-groups/quality-of-care/standards-for-improving-quality-of-maternal-and-newborn-care-in-health-facilities.pdf?sfvrsn=3b364d8_2.

[4] KIT Royal Tropical Institute, NSIA. Afghanistan Health Survey 2018. Kabul; 2019. https://www.kit.nl/wp-content/uploads/2019/07/AHS-2018-report-FINAL-15-4-2019.pdf.

[5] Berhan Y, Berhan A (2014). Antenatal care as a means of increasing birth in the health facility and reducing maternal mortality: a systematic review. Ethiop J Health Sci..

[6] Bauserman M, Lokangaka A, Thorsten V, Tshefu A, Goudar SS, Esamai F (2015). Risk factors for maternal death and trends in maternal mortality in low- and middle-income countries: a prospective longitudinal cohort analysis. Reprod Health..

[7] Wardle J, Steptoe A (2003). Socioeconomic differences in attitudes and beliefs about healthy lifestyles. J Epidemiol Community Health..

[8] Fagbamigbe AF, Idemudia ES (2017). Wealth and antenatal care utilization in Nigeria: policy implications. Health Care Women Int..

[9] Homan P, Brown TH, King B (2021). Structural intersectionality as a new direction for health disparities research. J Health Soc Behav..

[10] Akseer N, Bhatti Z, Rizvi A, Salehi AS, Mashal T, Bhutta ZA (2016). Coverage and inequalities in maternal and child health interventions in Afghanistan. BMC Public Health..

[11] Azimi MD, Najafizada SAM, Khaing IK, Hamajima N. Factors influencing non-institutional deliveries in Afghanistan: secondary analysis of the Afghanistan Mortality Survey 2010. Nagoya J Med Sci. 2015;77(1–2):133–43. PMC4361515 25797978

[12] Kim C, KMA S, Salehi AS, Zeng W. An equity analysis of utilization of health services in Afghanistan using a national household survey. BMC Public Health. 2016;16(1):1–11. 10.1186/s12889-016-3894-z PMC5139141 27919238

[13] Mumtaz S, Bahk J, Khang YH. Current status and determinants of maternal healthcare utilization in Afghanistan: analysis from Afghanistan demographic and health survey 2015. Plos One. 2019;14(6):e0217827. 10.1371/journal.pone.0217827 PMC6559709 31185028

[14] Shahram MS, Hamajima N, Reyer JA (2015). Factors affecting maternal healthcare utilization in Afghanistan: secondary analysis of Afghanistan Health Survey 2012. Nagoya J Med Sci..

[15] Afulani PA, Moyer C. Explaining disparities in use of skilled birth attendants in developing countries: a conceptual framework. Plos One. 2016;11(4):e0154110. 10.1371/journal.pone.0154110 PMC4841546 27105309

[16] Dahiru T, Oche OM. Determinants of antenatal care, institutional delivery and postnatal care services utilization in Nigeria. Pan Afr Med J. 2015;22(1). 10.11604/pamj.2015.21.321.6527 PMC4633744 26587168

[17] Gabrysch S, Campbell OMR (2009). Still too far to walk: literature review of the determinants of delivery service use. BMC Pregnancy Childbirth..

[18] Ryan BL, Krishnan RJ, Terry A, Thind A. Do four or more antenatal care visits increase skilled birth attendant use and institutional delivery in Bangladesh? A propensity-score matched analysis. BMC Public Health. 2019;19(1):1–6. 10.1186/s12889-019-6945-4 PMC6521440 31096959

[19] Bauer GR (2014). Incorporating intersectionality theory into population health research methodology: challenges and the potential to advance health equity. Soc Sci Med..

[20] Brown TH, Richardson LJ, Hargrove TW, Thomas CS (2016). Using multiple-hierarchy stratification and life course approaches to understand health inequalities: the intersecting consequences of race, gender, SES, and age. J Health Soc Behav..

[21] Cummings JL, Jackson PB (2008). Race, gender, and SES disparities in self-assessed health, 1974–2004. Res Aging..

[22] Kapilashrami A, Hankivsky O (2018). Intersectionality and why it matters to global health. Lancet..

[23] Schulz AJ, Mullings L. Gender, race, class, & health: Intersectional approaches. 2006.

[24] King DK (1988). Multiple jeopardy, multiple consciousness: the context of a black feminist ideology. Signs J Women Cult Soc..

[25] Veenstra G (2011). Race, gender, class, and sexual orientation: intersecting axes of inequality and self-rated health in Canada. Int J Equity Health..

[26] Rutstein SO, Johnson K. The DHS Wealth index, DHS comparative report 6. 2004.

[27] Rahman M, Saha P, Anwar N, Hossain A (2021). He hurts her womb: physical-sexual violence and pregnancy complications among women in Afghanistan. Heal Promot Perspect..

[28] Rajbanshi S, Norhayati MN, Nik Hazlina NH (2021). Perceptions of good-quality antenatal care and birthing services among postpartum women in Nepal. Int J Environ Res Public Health..

[29] Jallow IK, Chou YJ, Liu TL, Huang N (2012). Women’s perception of antenatal care services in public and private clinics in the Gambia. Int J Qual Heal Care..

[30] Anastasi E, Borchert M, Campbell OMR, Sondorp E, Kaducu F, Hill O (2015). Losing women along the path to safe motherhood: why is there such a gap between women’s use of antenatal care and skilled birth attendance? A mixed methods study in northern Uganda. BMC Pregnancy Childbirth..

[31] Choulagai B, Onta S, Subedi N, Mehata S, Bhandari GP, Poudyal A (2013). Barriers to using skilled birth attendants’ services in mid- and far-western Nepal: a cross-sectional study. BMC Int Health Hum Rights..

[32] Kibria GMA, Ghosh S, Hossen S, Barsha RAA, Sharmeen A, Uddin SMI (2017). Factors affecting deliveries attended by skilled birth attendants in Bangladesh. Matern Heal Neonatol Perinatol..

[33] Mengesha ZB, Biks GA, Ayele TA, Tessema GA, Koye DN (2013). Determinants of skilled attendance for delivery in Northwest Ethiopia: a community based nested case control study. BMC Public Health..

[34] Doctor HV, Nkhana-Salimu S, Abdulsalam-Anibilowo M (2018). Health facility delivery in sub-Saharan Africa: successes, challenges, and implications for the 2030 development agenda. BMC Public Health..

[35] Adewemimo AW, Msuya SE, Olaniyan CT, Adegoke AA (2014). Utilisation of skilled birth attendance in northern Nigeria: a cross-sectional survey. Midwifery..

[36] Pulok MH, Sabah MNU, Uddin J, Enemark U (2016). Progress in the utilization of antenatal and delivery care services in Bangladesh: where does the equity gap lie?. BMC Pregnancy Childbirth..

[37] Wilunda C, Quaglio G, Putoto G, Takahashi R, Calia F, Abebe D (2015). Determinants of utilisation of antenatal care and skilled birth attendant at delivery in South West Shoa Zone, Ethiopia: A cross sectional study. Reprod Health..

[38] Link BG, Phelan JC, Miech R, Westin EL (2008). The resources that matter: fundamental social causes of health disparities and the challenge of intelligence. J Health Soc Behav..

[39] Starfield B, Shi L. The medical home, access to care, and insurance: a review of evidence. Pediatrics. 2004;113(Supplement_4):1493–8. 15121917

[40] Ettner SL (1996). The timing of preventive services for women and children: the effect of having a usual source of care. Am J Public Health..

[41] Bengiamin MI, Capitman JA, Ruwe MB (2010). Disparities in initiation and adherence to prenatal care: impact of insurance, race-ethnicity and nativity. Matern Child Health J..

[42] Dey A, Hay K, Afroz B, Chandurkar D, Singh K, Dehingia N, et al. Understanding intersections of social determinants of maternal healthcare utilization in Uttar Pradesh, India. Plos One. 2018;13(10):e0204810. 10.1371/journal.pone.0204810 PMC6171889 30286134

[43] Sridharan S, Pereira A, Hay K, Dey A, Chandurkar D, Veldhuizen S (2018). Heterogeneities in utilization of antenatal care in Uttar Pradesh, India: the need to contextualize interventions to individual contexts. Glob Health Action..

[44] Montagu D, Yamey G, Visconti A, Harding A, Yoong J. Where do poor women in developing countries give birth? A multi-country analysis of demographic and health survey data. Plos One. 2011;6(2):e17155. 10.1371/journal.pone.0017155 PMC3046115 21386886

[45] Uddin J, Pulok MH, Johnson RB, Rana J, Baker E. Association between child marriage and institutional delivery care services use in Bangladesh: intersections between education and place of residence. Public Health. 2019;171:6–14. 10.1016/j.puhe.2019.03.014 31071578

[46] Kc S, Neupane S (2016). Women’s autonomy and skilled attendance during pregnancy and delivery in Nepal. Matern Child Health J..

[47] Moyer CA, Mustafa A (2013). Drivers and deterrents of facility delivery in sub-Saharan Africa: a systematic review. Reprod Health..

[48] Agha S, Carton TW. Determinants of institutional delivery in rural Jhang, Pakistan. Int J Equity Health. 2011;10(1):1–12. 10.1186/1475-9276-10-31 PMC3159141 21801437

[49] Tsegay Y, Gebrehiwot T, Goicolea I, Edin K, Lemma H, Sebastian MS (2013). Determinants of antenatal and delivery care utilization in Tigray region, Ethiopia: A cross-sectional study. Int J Equity Health..

[50] Kebede B, Gebeyehu A, Andargie G. Use of previous maternal health services has a limited role in reattendance for skilled institutional delivery: cross-sectional survey in Northwest Ethiopia. Int J Women's Health. 2013;5:79–85. 10.2147/IJWH.S40335 PMC3583437 23459063

[51] Roro MA, Hassen EM, Lemma AM, Gebreyesus SH, Afework MF (2014). Why do women not deliver in health facilities: a qualitative study of the community perspectives in south Central Ethiopia?. BMC Res Notes..

[52] Asefa A, Gebremedhin S, Messele T, Letamo Y, Shibru E, Alano A, et al. Mismatch between antenatal care attendance and institutional delivery in South Ethiopia: a multilevel analysis. BMJ Open. 2019;9(3):e024783. 10.1136/bmjopen-2018-024783 PMC6527994 30898814

