# Supplementary material for: Associations of antenatal care visit with utilization of institutional delivery care services in Afghanistan: intersections of education, wealth, and household decision-making autonomy
Source: BMC Pregnancy Childbirth. 2022 Mar 26;22:255. doi: 10.1186/s12884-022-04588-0 (PMC8961925; doi:10.1186/s12884-022-04588-0)
Supplement: Supplementary file 1 — Additional file 1. [file 12884_2022_4588_MOESM1_ESM.docx]

**Supplementary Table 1** Two-way interactions of ANC with wealth, education (in years), and decision-making autonomy for delivery at a health facility, and delivery assisted by a skilled birth attendant.

| Variables | Delivery at a health facility | Delivery assisted by a skilled birth attendant |
| --- | --- | --- |
|  | F Statistic | F Statistic |
| Wealth | 2.03** | 2.16** |
| Education (in years) | 3.11* | 4.15** |
| Decision-making autonomy | 2.88* | 4.23** |

*P<0.05; **p<0.001

**Supplementary Table 2** Adjusted odds ratios and 95% confidence intervals for institutional delivery care indicators from multilevel logistic regression (N= 15581).

| Number of ANC visits (Ref: 0) | Delivery at a health facility | | | | Delivery assisted by a skilled birth attendant | | |
| --- | --- | --- | --- | --- | --- | --- | --- |
|  | OR  (95% CI) | | OR  (95% CI) | OR  (95% CI) | OR  (95% CI) | OR  (95% CI) | OR  (95% CI) |
|  | Model 1 | | Model 2 | Model 3 | Model 1 | Model 2 | Model 3 |
| 1 | 3.72*  (3.13-4.41) | | 3.60*  (3.02-4.27) | 3.60* (3.03-4.28) | 3.74*  (3.14-4.45) | 3.62*  (3.04- 4.31) | 3.63*  (3.05-4.32) |
| 2 | 5.47*  (4.63-6.46) | | 5.25*  (4.45-6.20) | 5.24*  (4.44-6.19) | 5.65*  (4.79-6.65) | 5.43* (4.61-6.40_ | 5.43* (4.61-6.40) |
| 3 | 6.25*  (5.21-7.51) | | 6.05  (5.02-7.28) | 6.03  (5.01-7.26) | 6.20*  (5.16-7.45) | 6.02*  (5.0-7.3) | 6.02*  (5.0-7.26) |
| ≥4 | 8.65*  (7.24-10.33) | | 7.85* (6.57-9.39) | 7.80*  (6.53-9.33) | 9.87*  (8.2-11.8) | 8.97*  (7.47-10.78) | 8.90*  (7.42-10.69) |
| Random effects | | | | | | | |
| Cluster-level variance (SE) | | 1.26 (0.11)* | 0.94(0.07)* | 0.93(0.07)* | 1.24(0.10)* | 0.89 (0.07)* | 0.98 (0.06)* |
| Intra-class correlation (%) | 27% | | 22% | 22% | 27% | 22% | 22% |
| Goodness of fit criterion: | | | | | | | |
| AIC | 15254.23 | | 15025.17 | 15026.33 | 15106.13 | 14849.11 | 14847.82 |
| BIC | 15323.11 | | 15155.26 | 15171.73 | 15175.02 | 14979.23 | 14993.25 |

*P<0.05; ANC, antenatal care; CI, confidence interval; OR, odds ratio.

Model 1: Adjusted for respondents’ current age and place of residence.

Model 2: Further adjusted for education, wealth, and employment status.

Model 3: Further adjusted for decision-making autonomy and beating not justified index.
